# NSE, positively regulated by LINC00657-miR-93-5p axis, promotes small cell lung cancer (SCLC) invasion and epithelial-mesenchymal transition (EMT) process

**DOI:** 10.7150/ijms.58415

**Published:** 2021-10-15

**Authors:** Lin Lu, Zhiqiang Zha, Peiling Zhang, Dailing Li, Guolong Liu

**Affiliations:** Department of Medical Oncology, Guangzhou First People's Hospital, South China University of Technology, Guangzhou, Guangdong, China.

**Keywords:** NSE, LINC00657, EMT, Small cell lung cancer

## Abstract

**Background:** Neuron specific enolase (NSE) is a specific biomarker for SCLC. However, the biological roles and aberrant expression of NSE in SCLC have not been well illustrated.

**Methods:** The expression of NSE, miR-93-5p and LINC00657 in SCLC tissues and cell lines were detected using real time quantitative PCR (qRT-PCR) or immunohistochemistry. CCK8 assay was performed to detect cell proliferation. Cell migration and invasion capabilities were investigated by transwell assay. Epithelial-mesenchymal transition (EMT) process was verified by detecting epithelial marker E-cadherin and mesenchymal marker N-cadherin. The direct interactions between miR-93-5p and NSE or LINC00657 were predicted by bioinformatics tools and verified using dual luciferase reporter assay.

**Results:** Upregulated expression of NSE in SCLC tumor tissues were positively associated with advanced tumor stage, distant metastasis and poor overall survival. Overexpression of NSE promoted cell proliferation, migration, invasion and EMT in SCLC cells, while silence of NSE inhibited these effects. Mechanically, NSE expression was positively correlated with LINC00657, and negatively correlated with miR-93-5p. Moreover, NSE was positively regulated by LINC00657 through sponging of miR-93-5p. LINC00657 and miR-93-5p promoted SCLC cell migration, invasion and EMT by NSE-mediated manner.

**Conclusion:** Overall, our study revealed a novel role of NSE in SCLC. NSE was positively regulated by LINC00657 through competitively interacting with miR-93-5p, which may be potential targets for SCLC patients.

## Introduction

Small cell lung cancer, which accounts for 10-20% of lung cancer, is the most malignant lung cancer subtype 1, 2. SCLC is sensitive to chemotherapy and radiotherapy, but the patients are more prone to develop disease relapse and distant metastasis 3. The 5-year survival rate of SCLC is below 5% 4. In addition, few patients could get benefits from the targeted therapy and immunotherapy 5, 6. Moreover, the mechanisms underlying the aggressive behaviors and low clinical responses for immunotherapy haven't been elucidated. Therefore, understanding the underlying mechanisms is of great importance for identifying novel treatment targets for SCLC patients.

Neuron-specific enolase (NSE), also known as enolase 2 (NSE), is the most reliable biomarker in the diagnosis of SCLC, due to its high specificity 7, 8. Serum concentrations of NSE were positively correlated with larger tumor size, advanced tumor stage and distant metastasis 9. Besides, NSE demonstrated a promising role in predicting the chemotherapy and radiotherapy responses 10, 11. NSE could enhance aerobic glycolysis to promote survival and proliferation of tumor cells 12-14. NSE could be transferred to the cell surface, which resulted in activating survival promoting signal pathway to promote tumor cell migration 15. However, the biological functions and upstream regulatory factors of NSE have not been clearly defined in SCLC.

Long non-coding RNA (lncRNA), non-coding RNA longer than 200 nucleotides, demonstrated important roles in carcinogenesis 16, 17. LncRNAs could modulate various biological functions by regulating mRNA expression through sponging miRNAs 18, 19. In this study, we firstly studied the role of NSE in the migration, invasion and EMT process of SCLC. We further identified lncRNA/miRNA which could regulate the expression and function of NSE. Our study would provide novel insight into the metastasis and poor OS for SCLC patients.

## Material and methods

### Tissues specimens and Ethical statement

This study involved normal lung tissues and SCLC tissue samples. Ethics Committee of Guangzhou First People's Hospital approved this study. And all the patients were informed. Here, normal tissues were collected from the normal lung tissues surrounding tumors, which was histopathologically confirmed. All the cancer patients were histopathologically confirmed and none patients have received any prior anti-tumor therapies prior to the collection of the tissues. Patients were staged according to the AJCC) Cancer Staging Manual (seventh edition).

### Cell lines and cell culture

Human normal lung bronchial epithelial cell line 16HBE and SCLC cell lines (H446, H69, H209) were purchased from the Chinese Academy of Science. The cells were cultured in RMPI-1640 supplemented with 10% fetal bovine serum (FBS). All the cell lines were cultured in a humidified atmosphere of 37 °C containing 5% CO2.

### Real time quantitative PCR

Firstly, total RNA was extracted from tissue specimens and cell lines using TRIzol reagent as previously described 20, 21. Then complementary DNA was synthesized based on reverse transcription from total RNA using Takara cDNA Synthesis Kit. Resulting cDNA was subjected to real time quantitative PCR using SYBR green master mix. The sequences of the sense and antisense primers were as follows: 5'-AGCCTCTACGGGCATCTATGA-3' (F) and 5'- TTCTCAGTCCCATCCAACTCC-3' (R) for NSE;5'-ATTTTTCCCTCGACACCCGAT-3' (F) and 5'-TCCCAGGCGTAGACCAAGA-3' (R) for E-cadherin; 5'-AGCCAACCTTAACTGAGGAGT-3' (F) and 5'-GGCAAGTTGATTGGAGGGATG-3' (R) for N-cadherin; 5'-TGATAGGATACATCTTGGACATGGA-3' (F) and 5'-AACCTAATGAACAAGTCCTGACATACA-3' (R) for LINC00657; 5'-CTCCTCCTGTTCGACAGTCAGC-3' (F) and 5'-CCCAATACGACCAAATCCGTT-3' (R) for GAPDH; 5'-TTATGGGTCCTAGCCTGAC-3' (F) and 5'-CACTATTGCGGGCTGC-3' (R) for U6. Comparative threshold cycle (2-ΔΔCT) method was used to calculate the relative mRNA values. The relative expression levels of AP000695.2 were normalized to the value of GAPDH.

### Immunohistochemistry

Paraffin-embedded SCLC tissue sections from 68 cases of SCLC patients were collected at Guangzhou First People's hospital. After antigen retrieval, the slides were incubated with the primary antibody (NSE, 1:100, Abcam) overnight. Then diaminobenzidine (DAB) was used for immunohistochemical staining following the incubation with the secondary antibody. Then the slides were hematoxylin counterstaining, dehydrated and sealed. Finally, the images were collected under a microscope.

### Western blot

Total proteins from tissues specimens and cell lines were extracted using RIPA solution as previously described 20, 21. Briefly, total proteins were separated using SDS-PAGE and transferred to PVDF membrane. After being blocked by non-fat milk, the membranes were incubated with primary antibodies as follows: anti-NSE (Abcam, ab16808, 1:2000), anti-β-catenin (Proteintech, 66009-1-Ig, 1:5000), anti-E-cadherin (Proteintech, 20874-1-AP, 1:5000), and anti-N-cadherin (Affinity, AF4039, 1:1000). Then membranes were incubated with corresponding secondary antibodies and detected using the enhanced chemiluminescence system.

### Bioinformatic analysis

The interaction between LINC00657 and miR-93-5p were predicted using starBase v3.0 22. The interaction between miR-93-5p and NSE were predicted in miRBD 23, 24.

### Cell transfection

The cDNA sequence of NSE or LINC00657 was subcloned into the pcDNA3.1 expression vector (Invitrogen, Shanghai). Indicated H446 cells were transfected with miR-93-5p inhibitor or mimic (purchased from GenePharma) to knock down or overexpress miR-93-5p. Cells transfected with scramble was used as negative control (NC). All the transfection experiments were performed using Lipofectamine2000 (Invitrogen).

### Cell proliferation

Cell proliferation capability was assessed using Cell Counting Kit-8 (CCK8) assay. Briefly, 5000 cells in 100 μL culture medium were seeded in a 96-well plate. 10 μL CCK8 reagent was added and incubated at different time point (12, 24, 36, 48 h). The absorbance of each well was measured under a microplate reader at a wavelength of 450 nm.

### Cell migration and invasion

Cell migration and invasion capabilities were assessed using with or without pre-packed Matrigel (BD Bioscience, USA). Briefly, indicated cells suspended in serum-free medium were placed in upper chambers as a density of 1×10^5^ cells per chamber. After incubation for 12 h or 24 h, the cells in the upper chamber was removed, fixed with 4% paraformaldehyde and stained using 0.1% crystal violet. Cell migrated or invaded to the lower chamber were counted and photographed under an optical microscope.

### Dual-luciferase reporter assay

The sequences of target genes and mutation genes were synthesized by Sangon Biotech (Shanghai). LINC00657-wild type (-WT) plasmid and LINC00657-mutated (-mut) plasmid were co-transfected with NC or miR-93-5p mimic using Lipofectamine2000 (Invitrogen). Similarly, wild-type and mutant 3'-UTR of NSE were subcloned into the luciferase-containing vector pGL3-luciferase vector. Then the wt or mut reporter plasmid was co-transfected with NC or miR-93-5p mimic using Lipofectamine2000 (Invitrogen).

### Nuclear and cytoplasmic fractionation analysis

Nuclear and cytoplasmic fractions were separated according to the manufacturer's constructions (PARIS kit, Thermo Fisher Scientific, USA). Then qRT-PCR was performed to detect the expression levels of LINC00657 in the nuclear and cytoplasm. GAPDH and U6 were used as internal controls.

### Statistical analysis

GraphPad Prism 7.0 software was utilized for all statistical analysis. The relationships between NSE expression and various clinico-pathological parameters were assessed using chi-square test. The correlations between two groups were compared using Student's t-test, one-way ANOVA, or Pearson correlation coefficient analysis. A two-tailed p value < 0.05 was accepted as significant difference. These experiments are repeated at least 3 times.

## Results

### Upregulated NSE expression was correlated with poor survival of SCLC patients

**Figure 1:**
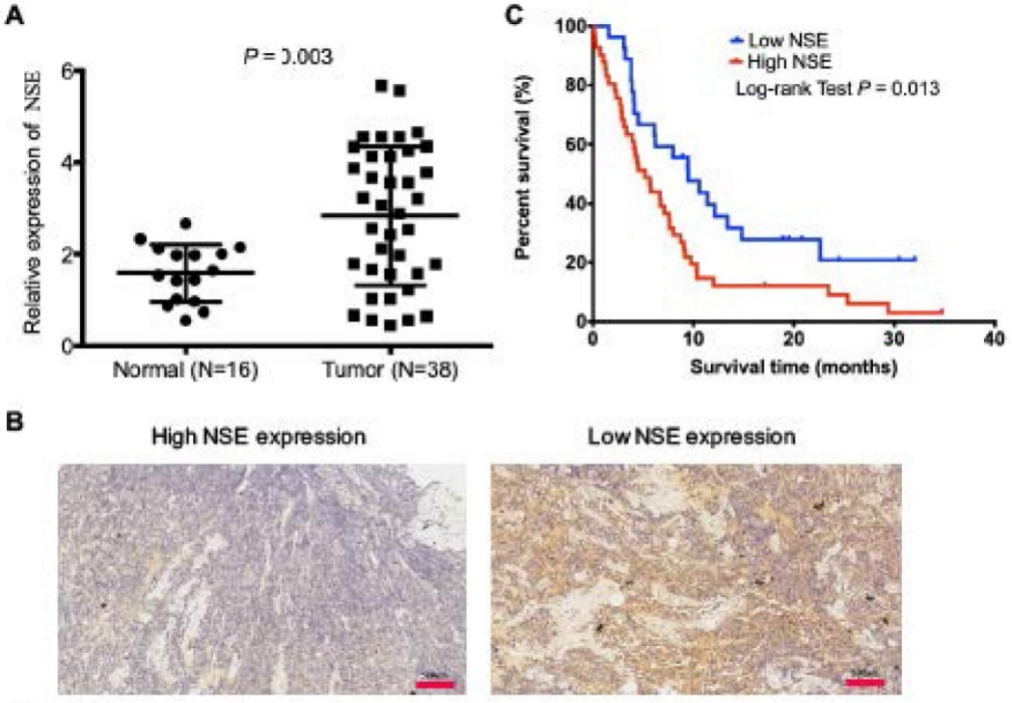
** Upregulated expression of NSE was positively correlated with poor overall survival of SCLC patients. (A)** Real time quantitative PCR (qRT-PCR) was performed to compare the expression of NSE in SCLC tumor tissues (N=38) and normal tissues (N=16). **(B)** The protein expression of NSE in SCLC tumor tissues were detected using immunohistochemistry (N=68). Representative images of high NSE expression and low NSE expression were demonstrated (magnification, x20). **(C)** Kaplan-Meier survival curve of the NSE expression in predicting overall survival (OS) of SCLC patients.

Serum NSE is an ideal biomarker for the diagnosis and treatment efficacy monitoring in SCLC. However, the expression of NSE in tumor tissues and its biological functions have not been well studied. In this study, we compared mRNA expression levels NSE between 16 cases of normal tissues and 38 cases of human SCLC tumor tissues. The qRT-PCR result showed that NSE expression was significantly upregulated in SCLC tissues (Figure 1A, *P* = 0.003). Immunohistochemistry confirmed the expression of NSE in SCLC tissues. Figure 1B demonstrated the representative microscopic images of NSE expression. Among the 68 cases of SCLC tissue sections, there were 52 cases (76.47%) demonstrated positive NSE expression. High NSE expression was positively correlated T stage (*P* = 0.025, Table 1), advanced TNM stages (*P* = 0.001, Table 1), as well as distant metastasis (*P* = 0.015, Table 1). Moreover, high expression of NSE was positively correlated with poor overall survival of SCLC patients (Figure 1C. *P* = 0.013). These data indicated that NSE acted as an oncogene, which might promote tumor progression and distant metastasis in SCLC.

### NSE promoted cell migration, invasion and EMT of SCLC cells

**Figure 2:**
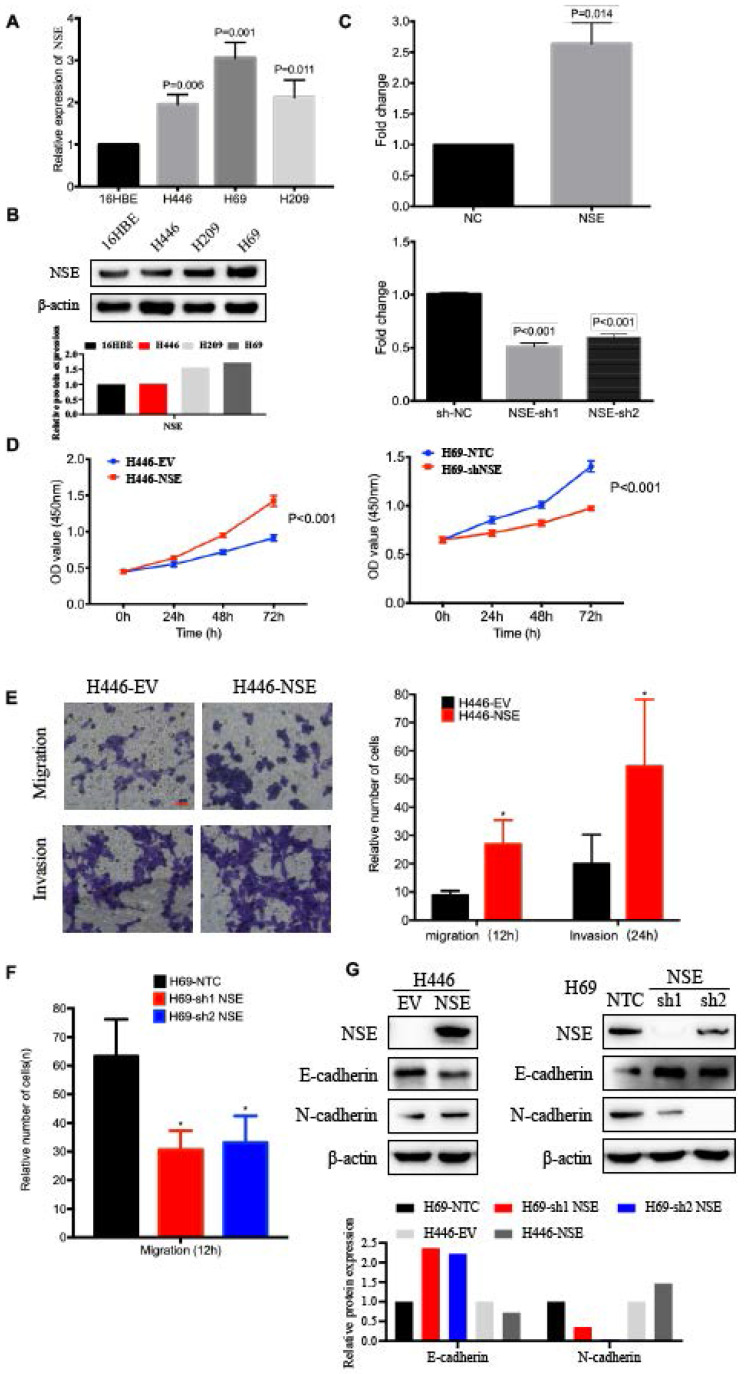
** NSE promoted the proliferation, migration and invasion of SCLC cells.** The relative mRNA expression levels of NSE in human bronchial epithelial cell 16HBE and various SCLC cell lines were detected using real time quantitative PCR (**A**).** (B)** The relative protein express levels of NSE were measured by western blot. And quantification of the relative protein amount was shown in the lower panel. **(C)** Real time quantitative PCR confirmed the expression of NSE after being overexpressed or knockdown. **(D)** The effect of NSE on the proliferation capabilities of SCLC cells were assessed using CCK8 assay. Transwell assay was performed to investigate the effect of NSE on the migration and invasion of SCLC cells. **(E)** Overexpression of NSE promoted cell migration and invasion of H446 cells. **(F)** Knockdown of NSE significantly inhibited cell migration. **(G)** Western blot results demonstrated overexpression of NSE promoted the EMT process by down-regulating the expression of E-cadherin and upregulating the expression of N-cadherin, while silencing NSE upregulated the expression of E-cadherin and downregulated the expression of N-cadherin. And quantification of the relative protein amount was shown in the lower panel.

The expression of NSE in human bronchial epithelial cell 16HBE and different SCLC cell lines were compared at mRNA and protein levels. As shown in Figure 2A and B, relative NSE expression was the highest in H69 and lowest in H446. Therefore, H69 and H446 were subjected to the subsequent cellular functions assays. To evaluate the biological function of NSE in SCLC, NSE overexpression and knockdown cells were constructed based on the differential expression of endogenous NSE expression. H446 cells were transfected with a lentivirus vector to construct NSE overexpression cells. H69 cells were transfected with short hairpin RNAs (shRNAs) to knock down endogenous NSE expression. The relative expression of NSE after overexpression or knockdown were verified using qRT-PCR (Figure 2C, *P* < 0.05).

The effect of NSE on cell proliferation was investigated using CCK8 assay. As shown in Figure 2D, overexpression of NSE promoted SCLC cell proliferation (*P* < 0.001), while knockdown of NSE inhibited cell proliferation (*P* < 0.001). Cell migration and invasion were investigated using transwell assay. Our results showed that NSE overexpression promoted H446 cell migration and invasion. The numbers of NSE overexpression cells migrated and invaded much more than control cells (Figure 2E). Knockdown of NSE significantly reduced the migration capability of H69 cells (Figure 2F).

Moreover, western blot results demonstrated that overexpression of NSE downregulated the expression of epithelial marker E-cadherin and upregulated the expression of mesenchymal makers N-cadherin. In contrast, knockdown of NSE upregulated the expression of E-cadherin and downregulated the expression of N-cadherin (Figure 2G). Taken together, these data indicated that NSE could promote the migration, invasion and EMT of SCLC cells.

### NSE expression was regulated by miR-93-5p

**Figure 3:**
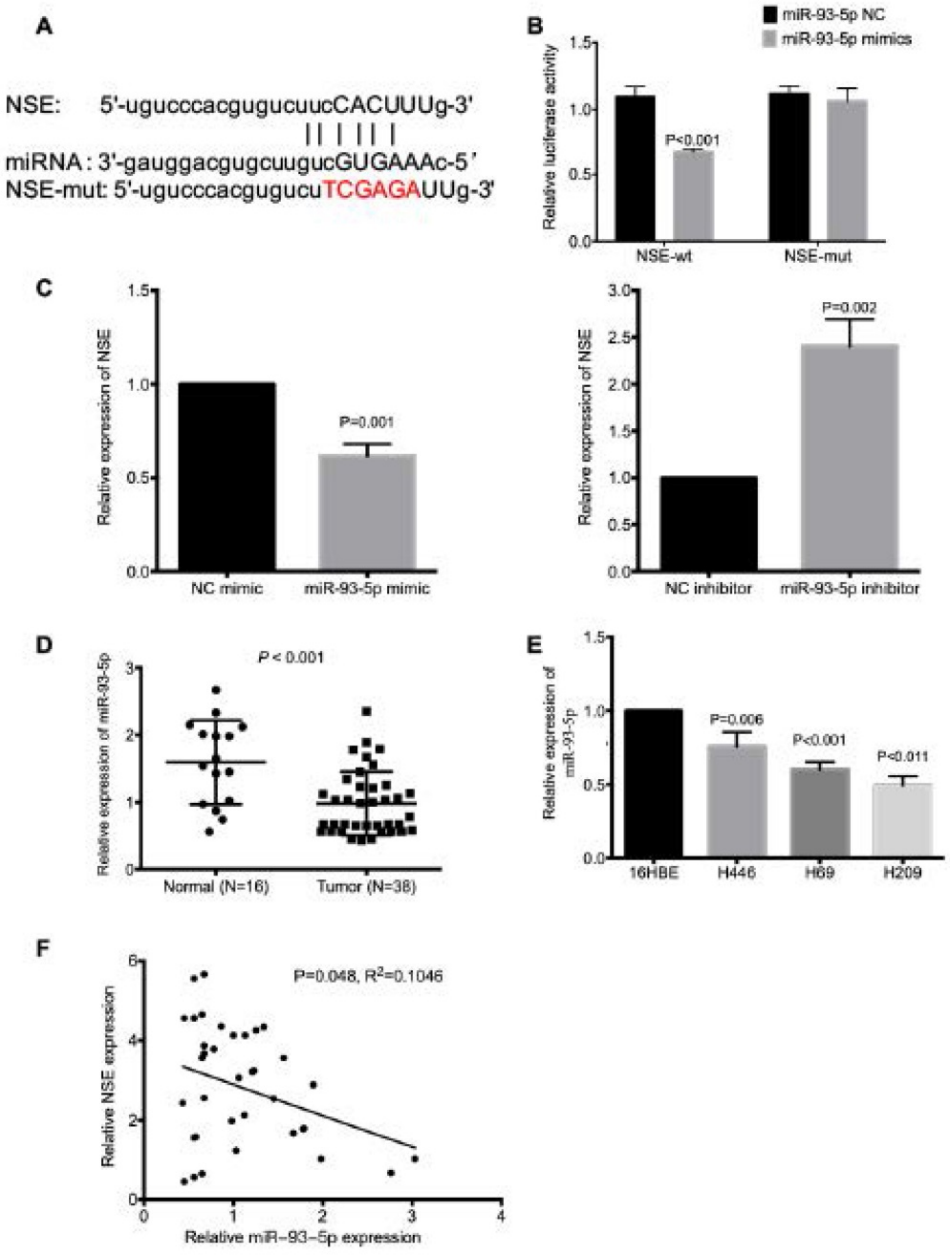
** NSE is a target of miR-93-5p in SCLC. (A)** The binding site between NSE and miR-93-5p was predicted by bioinformatics software. **(B)** Luciferase reporter assay confirmed the association between NSE and miR-93-5p. **(C)** Real time quantitative PCR demonstrated that miR-93-5p mimics downregulated the expression of NSE, while miR-93-5p inhibitor upregulated the expression of NSE. **(D)** qRT-PCR was performed to detect the expression of miR-93-5p between SCLC tumor tissues and normal tissues. **(E)** The expression of miR-93-5p in normal cell line 16HBE and SCLC cell lines were compared using qRT-PCR. **(F)** The negative correlation between NSE and miR-93-5p was compared using Pearson correlation coefficient in 38 SCLC tissues.

The mechanism underlying the upregulated NSE expression in SCLC is largely unknown, here we explored the miRNA which could modulate the expression of NSE. The miRNA-mRNA interactions database starBase (http://starbase.sysu.edu.cn/index.php) was applied to predict potential miRNAs that could interact with NSE. And we found that miR-93-5p could target NSE mRNA 3'UTR (Figure 3A). The interaction between miR-93-5p and NSE was validated using a dual luciferase reporter assay. As shown in Figure 3B, overexpression of miR-93-5p significantly decreased the luciferase activity of the NSE-wild type (NSE-WT) (*P* < 0.001), but not the luciferase activity of the NSE-MUT. In addition, the mRNA expression level of NSE was upregulated in cells transfected with miR-93-5p mimic (Figure 3C, *P* = 0.001). While miR-93-5p inhibitor increased the mRNA expression level of NSE (Figure 3C, *P* = 0.002). Moreover, the expression of miR-93-5p in SCLC tissues and normal tissues were assessed using qRT-PCR. We found significantly decreased expression of miR-93-5p in SCLC tumor tissues than in normal tissues (Figure 3D, *P* < 0.001). Higher expression of miR-93-5p were detected in SCLC cell lines while comparing with human normal lung bronchial epithelial cell line 16HBE (Figure 3E, *P* < 0.05). Moreover, Pearson correlation coefficient analysis demonstrated that there was negative correlation between NSE and miR-93-5p expression based on their mRNA expression levels in the 38 cases of SCLC specimens (Figure 3F, *P* = 0.048, R^2^=0.1046). These data indicated that miR-93-5p directly reduced the expression of NSE in SCLC cells.

### miR-93-5p inhibited cell migration, invasion and EMT of SCLC cell by down-regulated the expression of NSE

**Figure 4:**
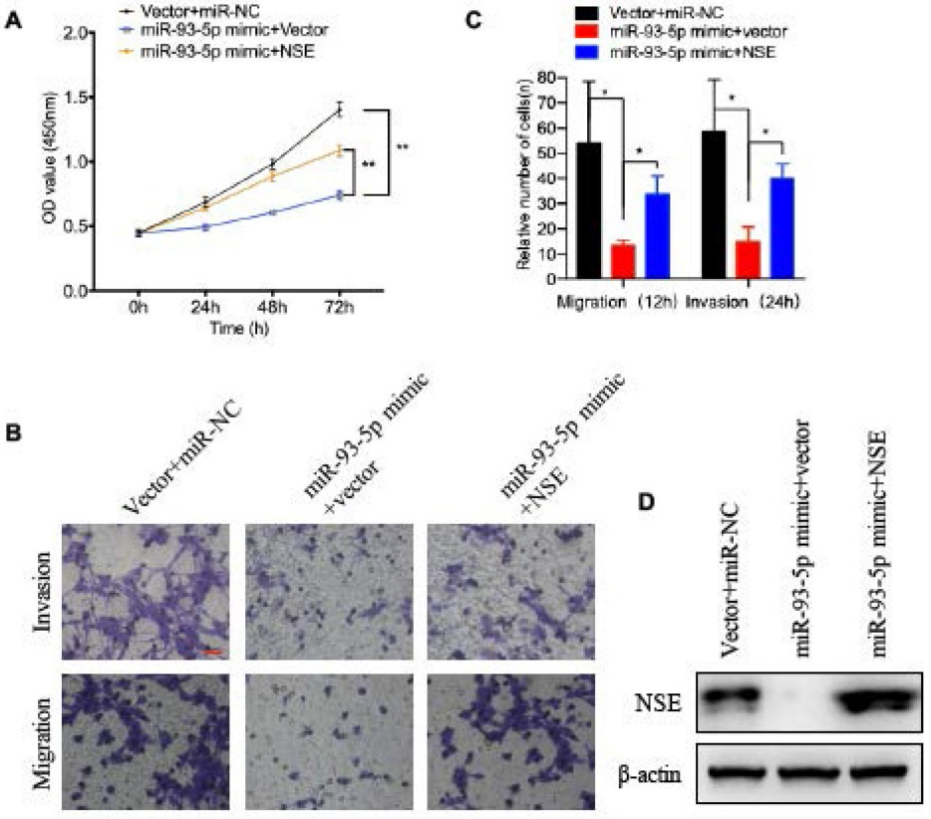
** The tumor suppressive capabilities of miR-93-5p was reversed by NSE overexpression.** SCLC cell line H69 was transfected with NSE overexpressed plasmids or/and miR-93-5p mimics. **(A)** Cell proliferation capabilities were assessed using CCK8 assay. **(B, C)** Cell migration and invasion capabilities were evaluated using transwell assay. **(D)** The expression levels of NSE in indicated cells were assessed using western blot analysis.

We next verified whether NSE was essential for the effect of miR-93-5p on the cellular functions of SCLC cells. NSE overexpressed cells were transfected with miR-93-5p mimic or control mimic. As shown in Figure 4A, overexpression of miR-93-5p significantly decreased the cell proliferation capabilities of SCLC cells. And overexpression of NSE partially abolished the inhibitory effect of miR-93-5p mimic on cell proliferation (Figure 4A, *P* < 0.01). Transwell assay demonstrated that treatment of miR-93-5p mimic significantly inhibited SCLC cell migration. And the inhibitory effect of miR-93-5p mimic on cell migration could be suppressed by NSE overexpression (Figure 4B). Similarly, invasion assay also demonstrated that the inhibitory effect of miR-93-5p mimic on cell invasion could be partially abolished by the overexpression of NSE (Figure 4B). Figure 4C shows the cell numbers migrated or invaded. These data indicated that miR-93-5p exerted tumor suppressive effects by downregulating NSE expression.

### LINC00657 acted as a sponge for miR-93-5p

**Figure 5:**
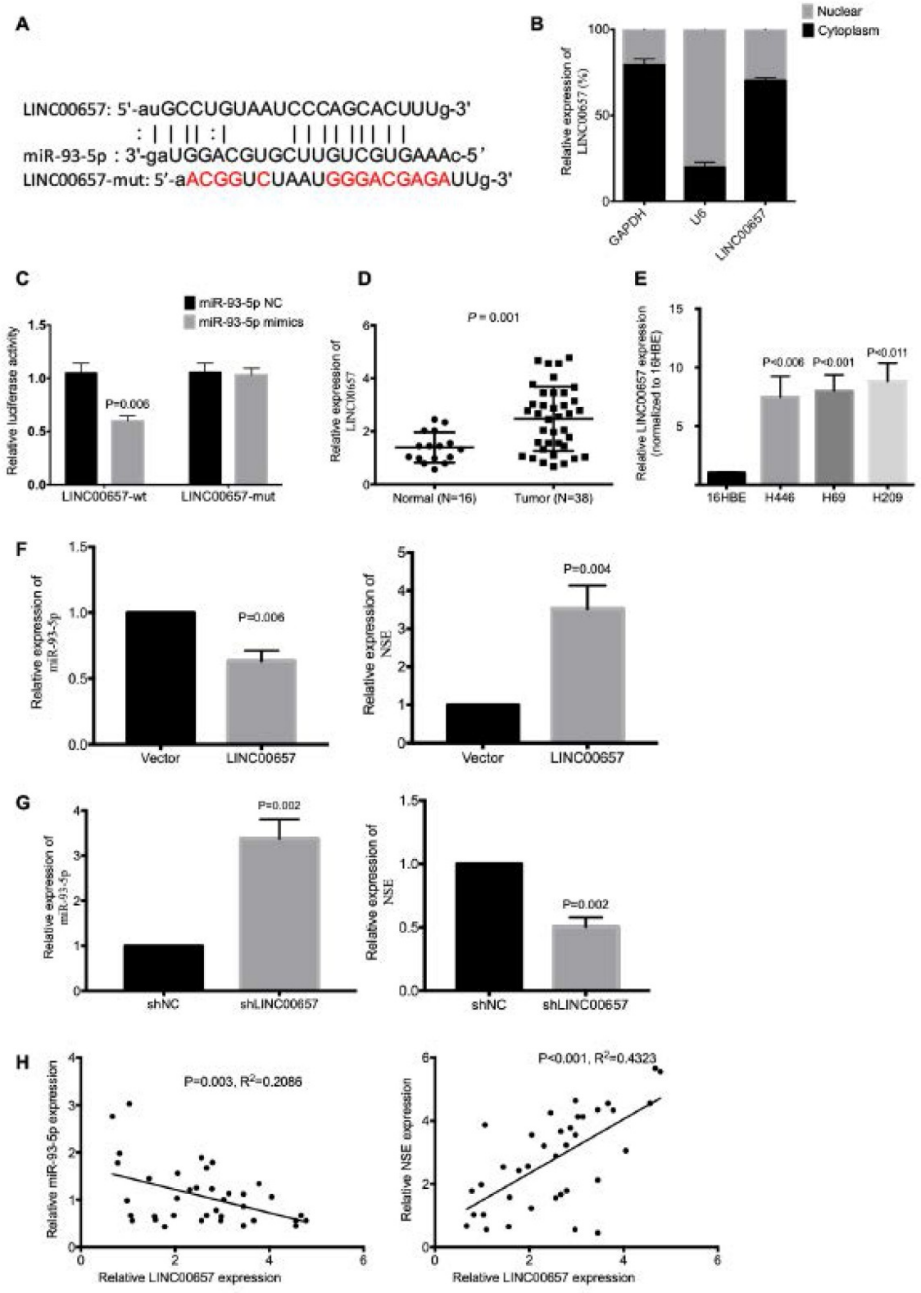
** LINC00657 acted as a sponge for miR-93-5p. (A)** Bioinformatics identified that LINC00657 could bind to miR-93-5p. **(B)** Cellular location of LINC00657 was detected using qRT-PCR. **(C)** Dual Luciferase reporter assay revealed the association between LINC00657 and miR-93-5p. **(D)** The expression of LINC00657 between SCLC tumor tissues and normal tissues were compared using qRT-PCR. **(E)** The expression of LINC00657 in normal cell line and SCLC cell lines H446, H69 was compared using qRT-PCR. The mRNA expression of miR-93-5p and NSE were assessed in LINC00657-overexpressed cells (**F**) and LINC00657 knockdown cells (**G**). **(H)** Pearson correlation coefficient revealed that there was negative correlation between LINC00657 and miR-93-5p. And there was positive correlation between LINC00657 and NSE. The data were based on the expression of LINC00657 and miR-93-5p in the 38 cases of SCLC tumor tissues.

We further explored the potential lncRNAs that could interact with miR-93-5p. According to starBase, we identified LINC00657 as a potential lncRNA (Figure 5A). Since cytoplasmic lncRNAs could work as ceRNA and regulate the target gene expression by sponging miRNA. We investigated the subcellular location of LINC00657 using nuclear-cytoplasm fractionation assay. The results showed that LINC00657 was mainly located in the cytoplasm of SCLC cells (Figure 5B). Wild-type (LINC00657-WT) and mutated (LINC00657-MUT) miR-93-5p binding sites were subcloned into dual-luciferase reporters. The luciferase assay results showed that miR-93-5p mimics significantly inhibited the relative luciferase activity of LINC00657-WT SCLC cells (*P* = 0.006), but exerted no effect on LINC00657-MUT cells (Figure 5C). The expression of LINC00657 in SCLC tissues and cell lines were detected using qRT-PCR. As shown in Figure 5D, LINC00657 expression was significantly upregulated in SCLC tumor tissues compared to normal tissues (Figure 5D, *P* = 0.001). And the expression of LINC00657 was much higher in SCLC cells than normal cell 16HBE (Figure 5E, *P* < 0.05). In addition, LINC00657 overexpression downregulated the expression of miR-93-5p (*P* = 0.006) and upregulated the expression of NSE (*P* = 0.004) (Figure 5F), while knockdown of LINC00657 upregulated the expression of miR-93-5p (*P* = 0.002) and downregulated the expression of NSE (*P* = 0.002) (Figure 5G). We further confirmed the correlation between LINC00657 and miR-93-5p based on the mRNA data generated from the 38 cases of SCLC tumor tissues. As shown in Figure 5H, Pearson correlation coefficient result showed that miR-93-5p expression was negatively correlated with LINC00657 (R^2^=0.2086, *P* = 0.003). Meanwhile, the expression of LINC00657 was positively correlated with the expression of NSE (R^2^=0.4323, *P* < 0.001). Taken together, these data indicated that LINC00657 might act as a sponge of miR-93-5p that directly target NSE in SCLC.

### LINC00657 modulated cell proliferation, migration, invasion and EMT of SCLC cells by regulating NSE

**Figure 6:**
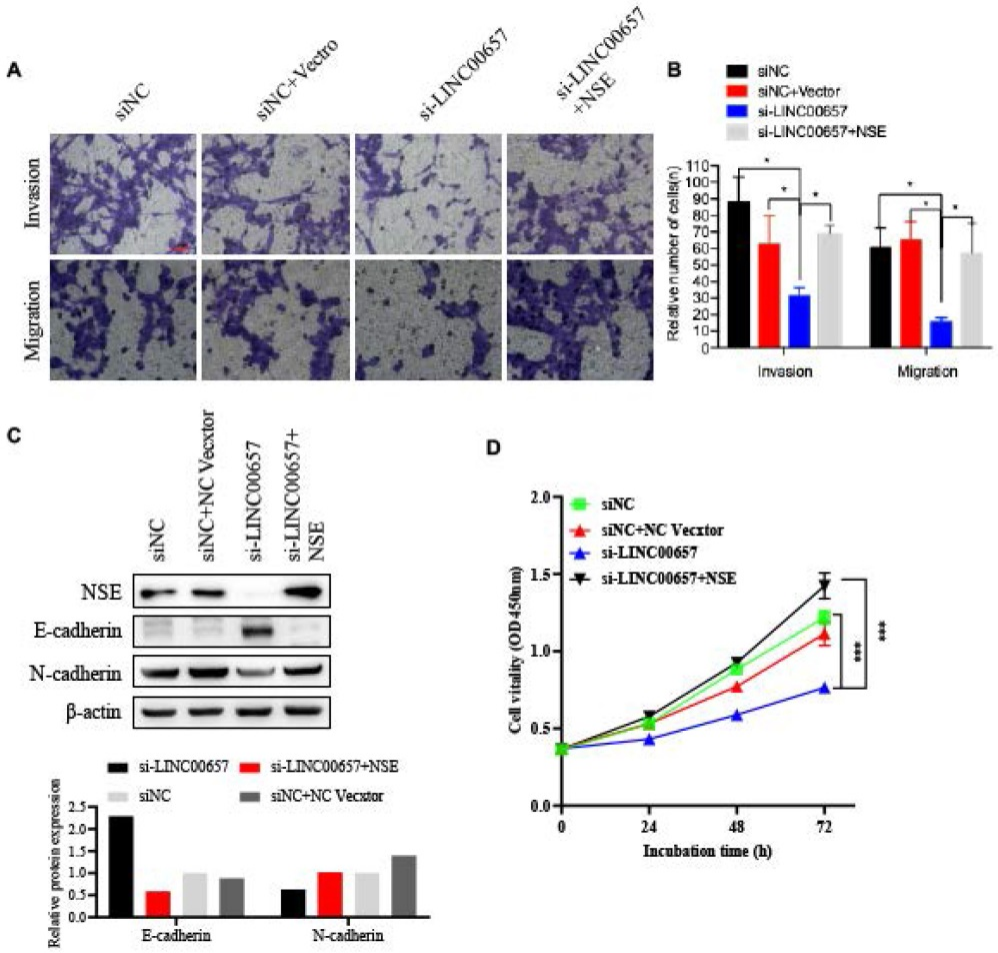
** LINC00657 promotes the migration and invasion of SCLC cells by upregulating the expression of NSE**. SCLC cell lung H69 was transfected with si-LINC00657 and/or NSE overexpression vector. (A, B) Cell migration and invasion capabilities were investigated using transwell assay. (C) The expression of NSE, EMT-related markers (E-cadherin and N-cadherin) were detected using western blot. And the quantification of the relative protein amount was shown in the lower panel. (D) Cell proliferation viabilities were assessed using CCK8 assay.

To further confirm the effect of LINC00657 in promoting the EMT process by regulating NSE, SCLC cell line H69 were transfected with siLINC00657 and/or NSE overexpression plasmid. Transwell assay indicated that silence of LINC0065 repressed SCLC cell migration and invasion. And the effect of LINC0065 on cell migration and invasion could be reversed by NSE overexpression (Figure 6A and B). Mechanically, silence of LINC00657 significantly upregulated the expression of E-cadherin and downregulated the expression of N-cadherin. And NSE overexpression could reverse the inhibitory effects of LINC0065 on the EMT process of SCLC cells, which was indicated by E-cadherin downregulation and N-cadherin upregulation (Figure 6C). In addition, NSE overexpression could reverse the effect of LINC0065 on cell proliferation of SCLC cells (Figure 5D). These results indicated that LINC00657 could modulate NSE to promote the migration, invasion and EMT of SCLC cells.

## Discussion

NSE is mainly expressed in neurons and neuroendocrine cells, which is generally accepted as a specific biomarker for neuroendocrine tumors, such as SCLC 25, 26. For a long time, most of the studies focused on the role of NSE as a biomarker in SCLC. Here, we discovered that upregulated expression of NSE was positively correlated with advanced tumor stage, distant metastasis and poor overall survival of SCLC patients. Epithelial-mesenchymal transition (EMT) is an important process for tumor cell invasiveness and metastasis initiation, by which tumor cells lose cell-cell adhesion and enable tumor cell invasion 27. Tumor cells gained EMT process largely contributed to tumor cell metastasis, gain of stem cell-like phenotype and drug resistance 28, 29. Here, our results indicated that modulation NSE expression could regulate SCLC cell migration, invasion and the expression of EMT makers, such as E-cadherin and N-cadherin. The modulation of NSE on EMT process might contribute to the enhanced migration and invasion capabilities of SCLC cells, as well as the distant metastasis. And our previous study also found that NSE could activate the wnt/beta-catenin pathway and promote the EMT process and tumor metastasis of SCLC cells 30. However, the mechanisms contributing to the upregulated expression of NSE in SCLC remain largely unknown.

miRNA-mRNA interactions could inhibit the translation or degrade targeted mRNAs to downregulated the expression of target genes 31, 32. The miRNA-gene interactions were involved in multiple cellular processes during tumor development, which including cell proliferation, cell migration, cell invasion, chemo-resistance, tumor relapse and distant metastasis 33-35. Moreover, miRNAs were correlated with chemoresistance, proliferation and poor outcome of SCLC such as miR-886-3p 36, miR-495 37. Thus, we next explored the potential miRNA which could interact with NSE and regulate the expression of NSE. According to bioinformatics analysis tool and dual luciferase reporter assay, we verified that miR-93-5p could directly interact with NSE. Downregulated expression of miR-93-5p was detected in SCLC tumor tissues and cell lines. There was a negative correlation between miR-93-5p expression and NSE expression. The relative expression levels of NSE were modulated by overexpression or knockdown of miR-93-5p. miR-93-5p, as a circulating miRNA, functioned as diagnostic or prognostic biomarker in multiple malignancies 38. Consistently, multiple studies also revealed that down-regulated expression of miR-93-5p was detected in tumor tissues and cell lines, such as colorectal cancer, gastric cancer 39-42.

There are multiple target genes for each miRNA 43. miR-93-5p could regulate HIF-1A/AXL signaling pathways to facilitate the progression and EMT of colorectal cancer 39. Liu et al found that downregulated expression of miR-93-5p could upregulate the expression of AHNAK to promote the progression of gastric cancer 41. Chen et al revealed that miR-93-5p functioned as a tumor suppressor by suppressing the expression of programmed death ligand 1 (PD-L1) 40. Here, we found that miR-93-5p inhibited cell proliferation, migration, invasion and EMT by directly targeting NSE.

Competitive endogenous RNA (ceRNA) regulatory network indicated the crosstalk between lncRNAs, miRNAs and mRNAs, in which mRNAs expression could be regulated by lncRNAs through sponging miRNAs 44, 45. Moreover, lncRNAs also have been found that was relevant to cellular proliferation, invasiveness, tumorigenesis and clinical relapse in SCLC such as lncRNA HOTAIR^46^, lncRNA H19^47^. Thus, we further explored lncRNAs which could interact with miR-93-5p to regulate the expression of NSE. Through bioinformatics analysis we found the potential interaction between miR-93-5p and LINC00657, which was further verified by dual luciferase assay. Overexpression of LINC00657 downregulated the expression of miR-93-5p and upregulated the expression of NSE. In contrast, knockdown of LINC00657 upregulated the expression of miR-93-5p and downregulated expression of NSE. Moreover, LINC00657 was mainly localized in cytoplasm and could be detected in exosomes 48. This suggested that LINC00657 could function as ceRNA. The role of LINC00657 in tumor development was controversial. Overexpression of LINC00657 repressed hepatocellular carcinoma cell growth 49. On the contrary, studies in colorectal cancer, breast cancer, oral squamous cell carcinoma, glioblastoma revealed that LINC00657 worked as an oncogenic lncRNA 50-52. Here, we found upregulated expression of LINC00657 in SCLC tumor tissues and cell lines. A positive correlation between NSE and LINC00657 expression levels was found in SCLC tumor tissues, while a negative correlation between NSE and miR-93-5p was observed.

Targeted mRNA expression could be regulated by lncRNAs/miRNA axis, which resulted in altering the biological activities of tumor cells. Moreover, the biological functions of LINC00657/ miR-93-5p/NSE in SCLC were further explored. Our results showed that overexpression of NSE abolished the inhibitory effect of LINC00657 on the migration, invasion and EMT of SCLC cells. Consistently, Zhang et al found that LINC00657 promoted the invasiveness and metastasis of osteosarcoma via regulating miR-106a and PD-L1 53. LINC00657 upregulated the expression of EMT-related target gene ZEB1 54. Bi et al. found that LINC00657 could act as a ceRNA by sponging miRNA-433 to upregulated PAK4 expression to promote invasion and progression of pancreatic ductal adenocarcinoma 55. Therefore, LINC00657, miR-93-5p and NSE formed regulatory axis for the EMT process regulation in SCLC.

The present study has several limitations. For example, we predicated the interaction of LINC00657 and miR-93-5p by starBase, but there is still a lack of direct evidence of physical interaction of LINC00657 and miR-93-5p.

In conclusion, proteins, miRNAs and lncRNAs all demonstrated promising noninvasive biomarkers for SCLC diagnosis and treatment surveillance. Here, we identified LINC00657/miR-93-5p/NSE in regulating the migration, invasion and EMT of SCLC. LINC00657 positively regulated the expression of NSE by sponging miR-93-5p in SCLC cells, thereby promoting EMT process, migration and invasion of SCLC cells. These results provided us with novel insights about the progression of SCLC. Our study will contribute to the exploring the biomarkers (LINC00657, miR-93-5p and NSE) for predicting prognosis and therapeutic targets in SCLC.

## Figures and Tables

**Figure 7:**
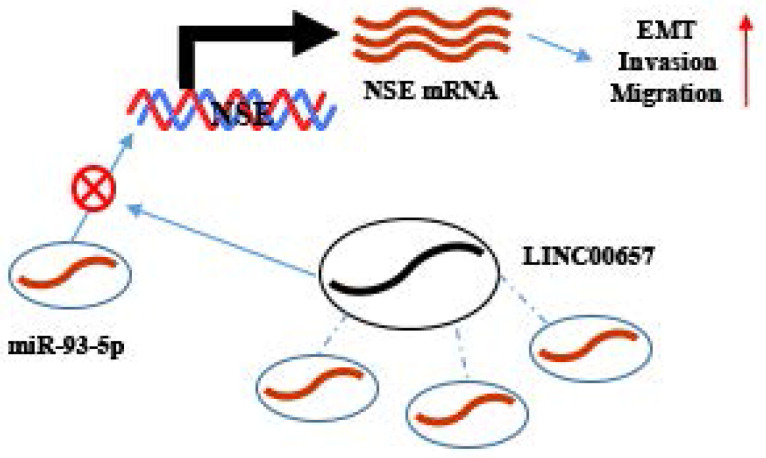
** Working model.** NSE was positively regulated by LINC00657 through sponging of miR-93-5p and promoted migration, invasion and EMT of SCLC cells.

**Table 1:** The relationships between NSE expression and clinicopathological parameters in SCLC

Characteristics	N	NSE		Χ^2^	*P-*value
		High expression	Low expression		
**Age**				0.793	0.373
≥60	30	22	8		
<60	38	24	14		
**Gender**				0.048	0.826
Male	42	28	14		
Female	26	18	8		
**Smoking**				1.635	0.201
Never-smoker	50	36	14		
Smoker	18	10	8		
**T stage**				5.029	0.025^*^
T1+2	33	18	15		
T3+4	35	28	7		
**N stage**				0.006	0.936
No	12	8	4		
Yes	56	38	18		
**M stage**				5.921	0.015^*^
M0	42	26	16		
M1	26	20	6		
**TNM stage**				10.76	0.001^*^
I+II	33	16	17		
III+IV	35	30	5		

## Abbreviations

NSE: Neuron specific enolase
SCLC: Small cell lung cancer
EMT: Epithelial-mesenchymal transition
qRT-PCR: Real time quantitative PCR
DAB: Diaminobenzidine
EV: Empty vector
